# Knowledge of tropical diseases and response capabilities of healthcare providers in Kaduna State, Nigeria

**DOI:** 10.1093/inthealth/ihad012

**Published:** 2023-04-21

**Authors:** David U Adje, Edmund D Dambo

**Affiliations:** Department of Clinical Pharmacy and Pharmacy Administration, Delta State University, Abraka, Nigeria; Health Systems Integration, FlyZipline International Nigeria, Kaduna, Nigeria

**Keywords:** capacity building, healthcare providers, knowledge base, neglected topical disease, Nigeria

## Abstract

**Background:**

The public health impact of neglected tropical diseases (NTDs) is quite substantial. The objective of this study was to assess the knowledge and response capability of health professionals regarding NTDs in Kaduna State, Nigeria.

**Methods:**

A pre-tested questionnaire with a Cronbach's α coefficient of 0.716 was administered to 350 health professionals. The questionnaire assessed the knowledge, resource availability and capacity to handle NTD cases.

**Results:**

Only 38 (12.6%) respondents were familiar with the World Health Organization's definition of NTDs. Although self-reported knowledge was highest for physicians (37 [82.2%]), there was no statistically significant knowledge disparity between cadres of health professionals. Only 12 (46.2%) practitioners in private health facilities reported adequate knowledge. The tier of practice was significantly associated with management of NTDs (χ^2^ = 10.545; df 2; p = 0.005). Only 24 (47.1%) medical laboratory scientists and 18 (40.0%) physicians had adequate clinical resources for management of NTDs. Nearly three-quarters (211 (70.1%)] of respondents had never been trained in the management of NTDs. More than half (177 [58.8%]) of facilities lacked pharmaceuticals or standard operating procedures for management of NTDs.

**Conclusions:**

Self-reported knowledge of NTDs was suboptimal. Physical and clinical resources for the diagnosis and treatment of NTDs were inadequate. Targeted training, increased funding and provision of adequate resources are needed in order to ameliorate the situation.

## Introduction

Neglected tropical diseases (NTDs) are a diverse group of diseases affecting the poorest 500 million people living in sub-Saharan Africa, Asia and the Americas. They are a significant cause of disability and reduced quality of life in affected persons.^1,2^ The impact of NTDs is worsened by resource limitations, poor infrastructure and weak health systems.^3^ In January 2012, the World Health Organization (WHO) published a roadmap with targets for eradication or elimination of the 17 NTDs. The 2020 target earmarked dracunculiasis and endemic treponematoses (yaws) for eradication while trachoma, Chagas disease, leprosy, lymphatic filariasis, rabies, onchocerciasis, schistosomiasis, leishmaniasis and human African trypanosomiasis were earmarked for elimination.^4^ Although some progress has been made, these targets have not been achieved due to a number of factors, including inadequate human and technical resources, unstable social and political environments resulting from conflicts and population displacement, resistance to medicines and pesticides, inadequate support for research and insufficient capacity to scale up interventions such as non-universal vector control measures.^5^

Nigeria, the Democratic Republic of Congo, Ethiopia and Tanzania contribute >50% of the NTD burden in Africa.^6^ It is therefore important to assess the country's human and material resource capability to achieve the WHO goal of eradication of NTDs using Kaduna State, northern Nigeria, as a case study.

The objectives of this study were to assess healthcare providers’ knowledge and familiarity with the WHO definition and classification of NTDs, to evaluate the human and material resources capabilities of Kaduna State in relation to achieving the goal of elimination or eradication of the WHO's NTDs and to determine the capacity of healthcare providers to provide specialized care and treatment to patients with NTDs in their facilities.

## Methods

### Setting

The study area was Kaduna State in the northern part of Nigeria (10°20′N 7°45′E). The state is bordered to the north by Kano State, to the northwest by Katsina State, to the south by Niger State and the Federal Capital Territory and to the east by Bauchi and Plateau States. There are 1583 registered healthcare facilities in Kaduna State, including 5 tertiary institutions, 30 secondary institutions, 1011 primary healthcare centres and 533 private health facilities.^7^

### Design

We used an exploratory cross-sectional survey design. Data were obtained using a pretested structured questionnaire designed by the authors based on a literature review. The questionnaire was administered to various cadres of heathcare professionals.

### Sample size and data collection

The geographic simple one-staged clustered sampling method was used for the study. The state was clustered into three mutually exclusive groups (Kaduna North, Kaduna Central and Kaduna South senatorial districts). The sampling unit was all population elements in the cluster. The sample size for the study was determined to be 341 using the Krejcie and Morgan table for statistical sample size determination, with the population size as 3106 health workers with a 95% confidence interval (CI) and a margin of error of 4.10%.^8^ Therefore a total of 350 questionnaires were distributed to randomly selected healthcare workers. A total of 120 health workers were randomly selected from the north and central senatorial districts and 110 from the south based on the population distribution of health workers in the state.

### Inclusion and exclusion criteria

All registered and practicing health professionals were included in the study. Health workers who were not actively practicing in registered public or private health facilities were excluded.

### Data collection

Pretesting was done using 20 healthcare workers from Barau Dikko Teaching Hospital, Kaduna State. Only respondents who expressed a willingness to participate by signing a written informed consent form were included in the study. Data were collected over a period of 3 months, from February to April 2021.

### Data analysis

Data analysis was performed using SPSS version 26.0 (IBM, Armonk, NY, USA).^9^ Responses were coded and inputted into SPSS software. Descriptive statistics were performed for all demographic and personal variables. Continuous variables were expressed as mean and standard deviation (SD) while categorical variables were expressed as frequencies and percentages. The relationship between variables of interest and demographics was explored using Pearson's χ2 tests where appropriate. A p-value <0.05 indicated statistical significance. Reliability testing for the data was done using the Cronbach's α coefficient.

## Results

A total of 350 questionnaires were administered, of which 301 were returned, giving a response rate of 86%. Overall reliability of the questionnaire was modest with a Cronbach's α coefficient of 0.719. Other dimensions explored also showed a good reliability profile. Knowledge of NTDs, management of NTDs, physical resources and clinical resources had Cronbach’s α coefficients of 0.672, 0.702, 0.699 and 0.790, respectively.

### Demographics of respondents

The majority of the respondents were from public health facilities (261 [90%]). Registered nurses had the greatest number of respondents (93 [30.9%]). More than one-quarter of health workers had practiced for more than 10 y (Table 1).

**Table 1.** Sociodemographic characteristics of healthcare workers (N=301)

Variable	Frequency	%
Tier of practice		
Tertiary health facility	38	12.6
Secondary health facility	216	71.8
Primary health facility	47	15.6
Profession of respondents		
Physician	45	15.0
Pharmacist	74	24.6
Registered nurse	93	30.9
Medical laboratory scientist	51	16.9
Community health worker	38	12.6
Practice specialty		
Specialist	13	4.3
Non-specialist	182	60.5
Not specified	106	35.2
Years of practice		
<2	51	16.9
2–5	90	29.9
5–10	66	21.9
>10	94	31.3

### Familiarity with and source of knowledge about NTDs

Only 38 (12.6%) of the respondents were familiar with the WHO definition and classification of NTDs. The predominant source of information about NTDs was the media and internet (93 [30.9%]), followed by seminars (93 [30.9%]), post-qualification training (74 [24.6%]) and pre-qualification training (45 [15%]).

### Self-reported knowledge of the WHO's NTDs

More than half (154 [59.0%]) of healthcare workers in public health facilities indicated that they had adequate knowledge of NTDs, compared with 12 (46.2%) from private health facilities. Self-reported knowledge was highest for physicians (37 [82.2%]), followed by medical laboratory scientists (36 [70.6%]), pharmacists (45 [60.8%]), registered nurses (52 [55.9%]) and community health extension workers (18 [47.3%]). However, differences in knowledge between the various cadres of health professionals was not statistically significant (Table 2).

**Table 2.** Relationship between self-reported knowledge of NTDs and demographic variables

Variable	Total	Frequency	%	χ^2^	df	p-Value
Type of practice						
Public health facility	261	154	59.0	1.601	1	0.206
Private health facility	26	12	46.2			
Tier of practice						
Tertiary health facility	38	25	65.8			
Secondary health facility	216	122	56.4	2.118	2	0.347
Primary health facility	47	31	66.0			
Profession of respondent						
Physician	45	37	82.2			
Pharmacist	74	45	60.8	6.506	4	0.164
Registered nurse	93	52	55.9			
Medical laboratory scientist	51	36	70.6			
Community health worker	38	18	47.3			
Practice specialty						
Specialist	13	11	84.6			
Non-specialist	182	52	28.6	3.046	3	0.385
Not specified	106	84	79.2			

### Health professional's experience with management of NTDs

One-quarter (75 [25%]) of respondents reported having managed one or more NTDs in the course of practice while only 14 (18.6%) had cases of NTDs referred to them. Only 16 (21%) respondents had referred cases of NTDs to other facilities. Almost half (20 [43.5%]) of the healthcare workers at the primary health facilities managed cases of NTDs, compared with 11 (32.4%) at tertiary health facilities and 44 (21.2%) at secondary health facilities. Physicians (16 [39.0%]) were most involved in the management of NTDs, followed by pharmacists (14 [20.0%]), registered nurses (24 [26.1%]), medical laboratory scientists (11 [22.9%]) and community health extension workers (10 [27.0%]). Only the tier of practice was significantly associated with management of NTDs (Table 3).

**Table 3.** Relationship between management of NTDs and demographic variables

Variable	Total	Frequency	%	χ^2^	df	p-Value
Type of practice						
Public health facility	261	65	25.0			
Private health facility	26	10	38.5	2.214	1	0.137
Tier of practice						
Tertiary health facility	38	14	36.8			
Secondary health facility	216	50	23.1	10.545	2	0.005
Primary health facility	47	23	48.9			
Profession of respondents						
Physician	45	21	46.7			
Pharmacist	74	19	25.7	5.177	4	0.270
Registered nurse	93	28	30.1			
Medical laboratory scientist	51	17	33.3			
Community health worker	38	12	31.6			
Practice specialty						
Specialist/not specified	119	76	63.9	5.321	3	0.150
Non-specialist	182	52	25.9			

### Availability of physical resources for management of NTDs

Nearly one-quarter (234 [77.7%]) of respondents had no dedicated clinic and 204 (67.8%) of the respondents did not have a focal person for NTDs in their facilities. The tier of practice and profession of respondents were significantly associated with the availability of physical resources (χ^2^=23.698, df 2, p=0.022; χ^2^=11.231, df 4, p=0.024, respectively) (Table 4).

**Table 4.** Relationship of physical resource availability with demographic variables^a^

Variable	Total	Frequency	%	χ^2^	df	p-Value
Type of practice						
Public health facility	261	34	13.5	10.946	1	0.071
Private health facility	26	10	38.5			
Tier of practice						
Tertiary health facility	38	9	23.7			
Secondary health facility	216	27	12.5	23.698	2	0.022
Primary health facility	47	21	46.7			
Profession of respondents						
Physician	45	6	13.3			
Pharmacist	74	16	21.6			
Registered nurse	93	22	23.7	11.231	4	0.024
Medical laboratory scientist	51	17	33.3			
Community health worker	38	7	18.4			
Practice specialty						
Specialist/not specified	119	67	56.3	1.871	3	0.600
Non-specialist	182	28	15.4			

a Physical resources: equipment, laboratory and diagnostics supplies, reagents and reference standards for confirmatory diagnosis.

### Availability of clinical resources for management of NTDs

More medical laboratory scientists stated they had clinical resources for management of NTDs (24 [47.1%]) compared with physicians (18 [40.0%]), registered nurses (31 [33.3%]), pharmacists (21 [28.4%]) and community health extension workers (8 [21.7%]). There was no statistically significant association between the availability of clinical resources and demographic variables (Table 5).

**Table 5.** Relationship of availability of clinical resources and demographic variables^a^

Variable	Total	Frequency	%	χ^2^	df	p-Value
Type of practice						
Public health facility	261	71	30.9	0.013	1	0.908
Private health facility	26	8	32.0			
Tier of practice						
Tertiary health facility	38	19	50.0			
Secondary health facility	216	50	23.1	15.098	2	0.051
Primary health facility	47	23	48.9			
Profession of respondents						
Physician	45	18	40.0			
Pharmacist	74	21	28.4			
Registered nurse	93	31	33.3	9.162	4	0.057
Medical laboratory scientist	51	24	47.1			
Community health worker	38	8	21.1			
Practice specialty						
Specialist	13	7	53.8	3.591	3	0.309
Non-specialist	182	53	29.1			
Not specified	106	72	67.9			

a Clinical resources: dedicated clinic, focal person and work tools.

## Discussion

Only about half of respondents reported a good knowledge of NTDs, which is comparable to the findings of other studies. A study conducted in Osun and Kwara States, Nigeria, found that 46.7% of clinicians had good knowledge of treatment and control measures for human schistosomiasis, while another hospital-based study carried out in Ibadan, Nigeria, reported an NTD knowledge score of 57.2%.^10,11^ Self-reported knowledge of NTDs was not significantly associated with the type of healthcare worker or practice specialty. Therefore, initiatives aimed at building capacity of healthcare providers in relation to NTDs should involve all types of health professionals.^12^

Of those who were familiar with the definition and classification of NTDs, half acquired such knowledge through the media, especially the internet. This agrees with other workers who have reported that the internet is the most consulted source of information and that retentive capacity of the knowledge acquired from the internet is 50% more when compared with other sources.^13^ The rest of the respondents acquired their knowledge from training schools and structured training in the course of practice. This indicates that formal training of health workers in the management of NTDs is either lacking or deficient and suggests a need for professional capacity development of the workforce beyond knowledge acquired from the media and internet.^14^

Even though self-reported knowledge of NTDs was highest among physicians, there was no statistically significant association between self-reported knowledge and the type of health worker. In contrast, Oladimeji et al.^15^ reported that regarding Ebola viral disease, doctors had the best knowledge scores (131/141 [92.9%], [95% CI 87.8 to 96.8]) compared with other healthcare professionals.

The poor knowledge base of healthcare workers with respect to NTDs might be attributed to the fact that less attention, focus and funding is given to the treatment and control of NTDs compared with other public health disease programs such as malaria, human immunodeficiency virus (HIV) and tuberculosis (TB), thereby limiting the involvement of healthcare workers in the management of NTDs.^16^ Also, the comparatively low level of funding for NTDs by development partners might be a critical factor contributing to the obscurity and consequent poor knowledge of health professionals.^17^

### Management of NTDs by respondents

The fact that three-quarters of the respondents had never managed at least one case of an NTD might be either due to a weak knowledge base or misdiagnosis.^18^ One-quarter of health workers either received a referred case of an NTD or referred a case of an NTD to another facility. The structure of healthcare delivery in Nigeria supports the referral of cases from clinics or hospitals with limited capacity to facilities of higher capacity.^18^ However, the referral practice and culture in Nigeria is quite weak, resulting in some health workers managing cases well beyond the capacity of their centres and their professional competence. Measures should therefore be put in place to strengthen the referral system for NTDs and possibly integrate the management of NTDs into the mainstream healthcare system in order to improve the quality of care for NTDs.^19,20^

There was a statistically significant relationship between the tier of practice and the management of NTDs. Health workers at the primary health facilities encountered more patients with NTDs than workers at the secondary and tertiary facilities. This is consistent with the fact that NTDs are found more among the rural poor and disadvantaged populations.^2,21^

### Availability of physical resources for management of NTDs

The non-availability of health management information system (HMIS) and logistics management information system (LMIS) tools as well as health commodities, as seen in this study, could seriously limit the capacity of trained personnel to treat and manage NTDs. It has been shown that appropriate work tools are a strong factor in productivity and achieving project and treatment outcomes.^22^ Therefore, provision of record tools and LMIS capacity building among healthcare workers is crucial in addressing NTD practice deficits.

There was no statistically significant relationship between the availability of physical resources and the type of health professional, practice specialty, type of practice and tier of practice of health workers. This might explain the low self-reported knowledge across types of health workers and tier of practice. The situation is further compounded by unfavourable government policies. For example, the Nigerian Supply Chain Integration Project in the Department of Food and Drugs in the Federal Ministry of Health has instituted integration of five program areas (malaria, HIV, TB, reproductive health and vaccines). The project was aimed at improving commodities management and strengthening the provision of tools for documentation and maintaining sustainable resources for supply chain management. The intervention was focused on improving disease outcomes and health indices. Even though many healthcare workers were trained in these programs, NTDs were not included in the first phase of the project. This neglect obviously may have negatively affected NTD capacity building for the Nigerian health workforce.^23^

### Availability **of** clinical resources for management of NTDs

Clinical resources are important in determining the capacity of health workers to deliver services to patients.^24^ In most facilities, NTD commodities and inventory control management tools were not available. Non-availability of commodities for the management of disease cases is one of the critical factors responsible for program failure.^23^ It is unfortunate that while other programs have strong implementing partner support along with good supply chain management plans, e.g. the last-mile delivery approach to ensuring commodities security for the priority programs (malaria, HIV and TB), NTDs have received comparatively less support. This trend is not just in Kaduna State or Nigeria, but it is a global phenomenon.^25^ In view of the gross lack of logistic and financial support, the appellate ‘neglected’ is truly justified.

Training is key in maintaining an up-to-date workforce and improving care and service delivery.^25,26^ Three-quarters of respondents have never received formal training in the management of NTDs. This situation negates prerequisites for optimum service delivery and emphasizes the need to fill this gap.^17^ Standard operating procedure (SOP) manuals ensure practice consistency and quality service delivery.^27,28^ The non-availability of such guiding documents reduces the quality of service delivery by healthcare workers, as can be demonstrated by the results of this study in Kaduna State.

In order to improve the knowledge and capacity of healthcare workers to respond to cases of NTDs, the Kaduna State Ministry of Health should support facilities to set up dedicated clinics for NTDs, with a focal person at least in all secondary and tertiary health facilities in the state. Also, structured training on the management of NTDs should be organized for healthcare workers in the system to improve service delivery. LMIS, HMIS and SOP manuals should be developed and provided to the facilities and monthly service data should be captured from such clinics alongside other programs such as malaria, HIV and TB. It is also important to strengthen the referral system for NTDs from communities through the three levels of the health system (primary to secondary to tertiary).^29^

### Strengths and limitations

The strength of this study lies in the focus on the capacity of healthcare professionals to manage NTDs and the availability of physical and clinical resources. These variables have hitherto not been properly explored. The study draws attention to a critical gap in the bid to eliminate or eradicate NTDs.

The limitations of this study include the fact that only five types of healthcare professionals were included in the study. Also, the information was obtained by self-report, which could have been subject to social desirability response bias. A more objective method of assessing knowledge, e.g. using a validated knowledge assessment tool, could have improved the validity and the generalization of these findings. Another limitation is the narrow scope of the study. Broadening the scope to include other states in the country could have increased the validity of generalizing the findings from this study.

## Conclusions

Self-reported knowledge of NTDs was suboptimal. The majority of healthcare professionals were not familiar with the WHO definition and classification of NTDs, as more than half did not know the number and names of the 13 NTDs found in the state. Physical and clinical resources (diagnosis, clinical care, pharmaceutical care, laboratory services and referral) to facilitate the provision of specialized care to patients with NTDs were inadequate. Also, the tools required by healthcare workers to deliver standard care to patients (dedicated clinic with a focal person, LMIS and HMIS data tools, SOP manuals and drugs) were either not available or inadequate in the majority of the facilities. These deficiencies have serious implications for the achievement of targets of the WHO roadmap and must be addressed if the goals are to be achieved. In addition to logistic support, donor support should focus more on health professional's capacity building through targeted training, as this study has revealed a huge gap in this area. Therefore, training activities need to be scaled up in order to derive the optimum benefits from donor support and intervention. It is also important that support for NTDs by various agencies be delivered in a more coordinated manner so as to ensure effectiveness. There is also a need for increased donor funding and provision of adequate resources for NTD programs.

Equally important is the need for countries to follow the NTD roadmap and integrate targets into national NTD master plans. National governments should also increase domestic funding of NTD eradication programs to complement donor support.

At the local level, NTD special treatment clinics, such as for HIV and TB, should be established in secondary health facilities to facilitate capacity development and better management of NTDs.

Studies to evaluate the level of implementation of the national NTD policy in other states with emphasis on leadership, governance and financing,(especially the extent of domestic funding for NTDs, are advised. Other aspects that could be explored in future studies include the need to assess the extent of integration of NTDs into the primary healthcare system and identifying specific deficits in data management tools and other resources for NTDs.

## Data Availability

The data supporting the findings of this study are available within the article.
